# Lightweight and Robust Radar Waveform Recognition Based on RepNRS-LPI-Net

**DOI:** 10.3390/s26175367

**Published:** 2026-08-25

**Authors:** Tianyu Liao, Jiwei Hu

**Affiliations:** School of Information Engineering, Wuhan University of Technology, Wuhan 430070, China; szyyty@126.com

**Keywords:** radar waveform recognition, re-parameterizable convolution, NRS-ECA, focal label smoothing loss, lightweight deployment

## Abstract

To address the degradation of low-probability-of-intercept (LPI) radar waveform recognition caused by noise dispersion and the masking of modulation-dependent structures in low-SNR Choi–Williams distribution (CWD) images, this paper proposes RepNRS-LPI-Net, an integrated framework for robust recognition and lightweight deployment. CWD converts each received waveform into a two-dimensional time–frequency image that characterizes temporal evolution, frequency variation, and localized energy distribution. The proposed RepDW block integrates 3 × 3, 1 × 3, and 3 × 1 depthwise branches with an identity branch during training to capture joint time–frequency, temporal-direction, and frequency-direction responses while preserving informative features. These branches are then algebraically fused for efficient deployment. In addition, NRS-ECA combines channel recalibration with channel-dependent soft shrinkage to attenuate weakly supported noise-like activations without assuming that all weak responses are noise. Focal modulation and label smoothing are conservatively adopted as auxiliary training strategies to address difficulty imbalance and confidence regularization. Experimental results show that RepNRS-LPI-Net achieves 79.553350% overall accuracy and 49.137529% low-SNR accuracy, while the deployment form reduces the parameter count to 37,142 and the learned-layer computation to 15,722,496 MACs. These results indicate that RepNRS-LPI-Net improves measured recognition performance while substantially reducing deployment complexity under the modeled multipath, Rayleigh-fading, Doppler, and AWGN conditions.

## 1. Introduction

With the rapid development of radar detection, electronic reconnaissance, target sensing, and electromagnetic spectrum confrontation technologies, radar signal recognition in complex electromagnetic environments has become an important task in electronic support measure systems and cognitive electronic warfare systems. Low-probability-of-intercept (LPI) radars reduce the probability of detection by non-cooperative receivers through low radiated power, wideband modulation, frequency agility, phase coding, and complex intrapulse modulation. As a result, they have been widely used in target detection, threat warning, battlefield situational awareness, and electronic countermeasure scenarios [1]. Further, electronic reconnaissance systems are required to perform parameter estimation, intrapulse modulation analysis, and emitter identification on intercepted radar signals to support subsequent threat assessment and countermeasure decision-making [2]. However, the low-intercept properties of LPI radar waveforms are usually achieved through more complex signal structures and more flexible modulation schemes. Although these properties enhance the concealment and survivability of radar systems, they also increase the difficulty of automatic waveform recognition at the intercept receiver. In particular, under complex propagation conditions such as low signal-to-noise ratio (SNR), multipath propagation, noise interference, and channel fading, the discriminative differences among different modulation waveforms may be weakened, and the time–frequency structures of some waveforms may overlap. This can lead to degraded recognition performance for LPI radar waveforms. Therefore, investigating robust and lightweight LPI radar waveform recognition methods that can adapt to low-SNR and complex propagation environments is of both theoretical significance and practical engineering value.

Early studies on LPI radar waveform recognition mainly relied on signal-processing-based features and traditional machine learning classifiers. These methods typically design handcrafted features according to the physical structures and statistical properties of radar signals, and then employ classifiers for waveform recognition. Typical features include instantaneous frequency, instantaneous phase, autocorrelation function, power spectral density, higher-order moments, higher-order cumulants, and envelope statistics. Zhang et al. investigated LPI radar waveform recognition under strong-noise conditions by extracting signal features from time–frequency distributions and feeding them into a classifier, demonstrating the effectiveness of time–frequency analysis for representing non-stationary radar waveforms [3]. Kishore and Rao studied automatic intrapulse modulation classification for advanced LPI radar waveforms and showed that instantaneous parameters and statistical features can enhance the separability among different modulation types [4]. Compared with directly processing one-dimensional time-domain waveforms, these methods transform complex modulation structures into physically meaningful feature representations and have therefore received considerable attention in early research on LPI waveform recognition.

In traditional machine learning frameworks, combining time–frequency images with shallow classifiers is also an important recognition strategy. Since LPI radar signals are typically non-stationary, time–frequency analysis can simultaneously characterize temporal and frequency variations, making it more suitable than single-domain time or frequency features for representing complex waveforms such as frequency-modulated, phase-coded, and polyphase-coded signals. Bektaş and Ergezer generated time–frequency images based on the Choi–Williams distribution (CWD), extracted principal components from these images, and then used binary and multiclass SVM classifiers for LPI radar waveform recognition, thereby improving recognition accuracy [5]. Willetts et al. compared the performance of different time–frequency distributions in LPI radar pulse classification and pointed out that the resolution and SNR dependence of time–frequency representations vary across different signal parameter estimation tasks [6]. These studies indicate that traditional methods have gradually evolved from one-dimensional handcrafted feature-based approaches to frameworks that combine time–frequency representations with machine learning classifiers.

Related signal-processing research optimizes radar-embedded communication waveforms and studies radar/communication design trade-offs. Blunt et al. formulated performance characteristics and metrics for intra-pulse radar-embedded communication, while Ciuonzo et al. used multiobjective optimization to balance competing waveform design criteria [7,8]. These investigations concern transmitted-waveform design and embedded communication rather than received LPI waveform classification; they therefore provide complementary background, not recognition baselines for the present experiments.

Although traditional methods can achieve satisfactory recognition performance under certain conditions, they still have several limitations. First, handcrafted features strongly depend on expert knowledge and parameter settings, and their generalization capability is limited when waveform parameters change, noise intensity increases, or propagation conditions vary. Second, shallow classifiers such as SVM are sensitive to the quality of input features and are vulnerable to noise perturbations under low-SNR conditions. Third, traditional recognition pipelines usually involve multiple stages, including feature design, feature selection, and classifier training, which makes it difficult to achieve end-to-end adaptive feature learning. As dataset scales increase and waveform types become more diverse, relying solely on handcrafted features and shallow models is no longer sufficient to meet the requirements for robust and intelligent LPI radar waveform recognition in complex electromagnetic environments.

In recent years, deep learning has become an important research direction for LPI radar waveform recognition. Compared with traditional methods, deep neural networks can automatically learn hierarchical features from input data, reduce the dependence on handcrafted feature design, and enhance the representation capability for complex waveform patterns through nonlinear mapping. Kong et al. applied convolutional neural networks (CNNs) to automate LPI radar waveform recognition, in which discriminative features were learned from input signal representations, demonstrating the feasibility of deep learning for this task [9]. Subsequently, Ma et al. introduced neural architecture search into LPI radar waveform recognition and adopted differentiable architecture search to automatically design classifiers for multiple LPI radar waveform types, reducing the dependence on manually designed CNN backbones and improving the adaptability of deep models to radar waveform recognition tasks [10]. These studies indicate that deep-learning-based LPI radar waveform recognition has gradually evolved from directly applying conventional CNN structures to automatically optimizing network architectures for more robust waveform representation.

Following the initial progress achieved by CNN-based frameworks, researchers have further improved recognition performance from the perspectives of feature fusion, multi-branch representation, and semi-supervised learning. Quan et al. proposed a dual-channel CNN-based LPI radar signal recognition method, in which CWD time–frequency images are used as inputs, and handcrafted contour features and deep image features are jointly extracted and fused to improve recognition accuracy under low-SNR conditions [11]. More recently, Liao et al. explored semi-supervised learning for LPI radar waveform recognition by using a mean-teacher-based framework to exploit unlabeled samples and enhance model generalization when labeled data are limited [12]. Compared with methods that use a single time–frequency image and a fixed CNN classifier, these studies indicate that feature fusion, architecture optimization, and semi-supervised representation learning can provide richer discriminative information for deep models and further improve the recognition of complex LPI radar waveforms.

To mitigate severe noise contamination in time–frequency images under low-SNR conditions, several studies have further improved recognition performance from the perspectives of denoising enhancement, feature-space constraints, and metric learning. Jiang et al. proposed an improved LPI radar waveform recognition framework that combines LDC-Unet and SSR-Loss, in which a locally densely connected U-Net is used to enhance time–frequency image features and a noise-robust loss function is adopted to improve recognition performance [13]. Kim et al. proposed a patch-based noise reduction method to alleviate the influence of noise on LPI radar time–frequency image recognition [14]. Liu and Li proposed a radar signal recognition method based on a triplet convolutional neural network, in which triplet loss is used to optimize the feature-space distribution and enhance intra-class compactness and inter-class separability [15]. In addition, Ren et al. proposed an LPI radar signal recognition method based on feature enhancement and deep metric learning, thereby improving recognition capability under noise interference [16]. These studies show that low-SNR recognition can be improved not only through input-level denoising but also through feature enhancement, loss-function constraints, and metric learning [17].

In addition to noise-robust learning, network architecture optimization is another important direction in LPI radar waveform recognition. Wang et al. proposed an ACDCA-ResNeXt method to enhance LPI radar signal modulation recognition by improving the residual structure and attention mechanism [18]. Chen et al. proposed an improved ResNeSt-based method for LPI radar signal modulation recognition in complex multipath environments, aiming to improve recognition performance under complex propagation conditions [19]. Luo et al. applied ConvNeXt and Focal Loss to radar waveform recognition, using a stronger backbone network and a hard-sample weighting strategy to improve the recognition of complex multi-class waveforms [20]. Qi et al. proposed an improved EfficientNet-based LPI radar waveform modulation recognition method, in which an attention mechanism and Focal Loss are introduced to improve recognition performance under low-SNR conditions [21].

In summary, existing methods have effectively advanced automatic LPI radar waveform recognition, but several challenges remain. First, under low-SNR and complex propagation conditions, noise-induced responses may interfere with the extraction of effective time–frequency features, leading to degraded recognition accuracy. Second, some deep learning models rely on complex network architectures to improve recognition performance, which increases the number of parameters, model size, and inference overhead, thereby limiting their deployment on resource-constrained platforms. Therefore, achieving a better trade-off among low-SNR robustness, model compactness, and deployment efficiency remains an important problem worthy of further investigation.

To address these issues, this paper develops RepNRS-LPI-Net as a task-oriented co-design for low-SNR LPI waveform recognition and lightweight deployment. CWD images expose modulation-dependent ridges, coding transitions, temporal evolution, and frequency variation, but low SNR produces dispersed and weakly structured responses that can obscure these cues. RepDW consequently uses depthwise 1 × 3, 3 × 1, and 3 × 3 branches for temporal-direction, frequency-direction, and joint local structures, together with an identity branch that preserves informative responses. NRS-ECA combines local channel interaction with adaptive soft shrinkage so that weakly supported, noise-like activations are attenuated while discriminative channels are recalibrated. Focal modulation with label smoothing is treated as an auxiliary training objective rather than a standalone architectural novelty. After training, exact two-stage algebraic fusion produces a compact deployment graph. The contributions are summarized as follows:

- A task-oriented RepDW-based recognition architecture is developed for directional feature extraction from CWD images and exact train-to-deploy re-parameterization. The design combines depthwise lightweight filtering, training-time multi-branch representation, and a compact fused deployment graph.
- An NRS-ECA module is integrated to couple channel recalibration with channel-dependent soft shrinkage for low-SNR feature enhancement. The mechanism uses global and localized response statistics while avoiding the assumption that all low-amplitude responses are noise.
- Comprehensive validation is provided using a fixed independent dataset, controlled RepDW/NRS-ECA/loss ablations, exact complexity accounting, fusion equivalence, deployment latency, and subgroup analyses for the modeled Doppler, multipath, and Rayleigh-fading variations. Focal modulation and Label Smoothing are assessed as training strategies rather than claimed as a fundamentally new loss.

## 2. LPI Radar Signals and CWD Time–Frequency Representation

### 2.1. LPI Radar Signal Model

In an electronic reconnaissance receiver chain, the intercepted LPI radar signal is not an ideal waveform, but rather an observation affected by the combined effects of the propagation channel, noise, and multipath propagation. Let $x\left(k\right)$ denote the ideal transmitted waveform, $h\left(k\right)$ denote the equivalent channel response, and $n\left(k\right)$ represent complex Gaussian white noise. The received sequence can then be expressed as

(1) $$y\left(k\right)=x\left(k\right)⊗h\left(k\right)+n\left(k\right)$$

where k denotes the sampling index and ⊗ represents the convolution operation. If the original sampled sequence is real-valued, the Hilbert transform can be used to construct its analytic signal, thereby preserving amplitude, phase, and frequency variation information simultaneously:

(2) $$y^{\prime }\left(k\right)=y\left(k\right)+jH\left[y\left(k\right)\right]$$

This study focuses on 13 representative LPI radar waveforms, including linear frequency modulation (LFM), rectangular pulse (Rect), Costas code, Barker code, Frank code, P1, P2, P3, P4, and the time-coded waveforms T1, T2, T3, and T4. These waveforms exhibit distinct modulation characteristics under ideal conditions. However, in low-SNR and multipath environments, some waveforms may present similar energy distributions or overlapping trajectories in the time–frequency domain, making their time–frequency textures difficult to distinguish. Therefore, a more robust waveform recognition model is required.

### 2.2. Choi–Williams Time–Frequency Analysis

To convert one-dimensional radar signals into two-dimensional representations suitable for convolutional neural networks, the Choi–Williams distribution (CWD) is adopted to generate time–frequency images. CWD is an improved Cohen-class time–frequency distribution. Its main characteristic is the introduction of an exponential kernel function during time–frequency analysis, which suppresses the cross-terms commonly observed in the Wigner–Ville distribution while preserving a relatively high time–frequency resolution and reducing artifact interference. The CWD of the received signal y can be expressed as

(3) $${CWD}_{y}\left(t,\omega \right)=\Phi \left(\theta ,\tau \right)A_{y}\left(\theta ,\tau \right)exp\iint \left[j\left(\theta t-\tau \omega \right)\right]d\theta d\tau$$

(4) $$\Phi \left(\theta ,\tau \right)=\exp\left(-\frac{\theta^{2}\tau^{2}}{\sigma }\right)$$

where t and ω denote the time variable and angular frequency variable, respectively. The variables θ and τ denote the frequency-lag and time-lag variables, respectively. $A_{y}\left(\theta ,\tau \right)$ represents the ambiguity function of the signal, and σ is the scale factor of the kernel function. After the CWD transformation, different modulation types exhibit distinct energy trajectories, local slopes, frequency-hopping patterns, or phase-coding textures in the resulting images. Compared with directly processing one-dimensional sequences, two-dimensional time–frequency images can more intuitively represent the time-varying frequency structures of LPI waveforms and are more suitable for subsequent local texture learning by neural networks.

### 2.3. Dataset Construction and Preprocessing

The dataset contains 13 waveform classes and 31 integer SNR levels from −20 to +10 dB at 1 dB intervals. For every (class, SNR, realization index), 1000 samples were generated as independent physical realizations, each with independently drawn active waveform parameters, clean waveform, channel realization, Doppler value, and AWGN; a realization index is not reused physically across SNR levels. The 403,000 samples were split independently within each class × SNR stratum into 700 training, 150 validation, and 150 test realizations, yielding 282,100/60,450/60,450 samples. No waveform/channel/noise realization crosses split boundaries. Each test SNR therefore has 13 × 150 = 1950 samples. Low SNR is −20 to −10 dB (11 levels; 21,450 tests), mid SNR is −9 to 0 dB (10 levels; 19,500), and high SNR is +1 to +10 dB (10 levels; 19,500).

Each clean waveform is passed through a six-path Rayleigh channel comprising one zero-delay path and five independently parameterized nonzero-delay paths. The nonzero delays are sampled from 1 ns to 1 microsecond, average path gains from −20 to 0 dB, and maximum Doppler shift from 10 to 1000 Hz; the channel uses a Jakes spectrum and is reset for each independent realization. Complex circular AWGN is then added at the designated SNR. Accordingly, the evaluation supports claims only for the modeled multipath, Rayleigh-fading, Doppler, and AWGN conditions; explicit synchronization mismatch was not tested.

The channel-plus-noise waveform is transformed using the FTCWD implementation, linearly mapped per image to 0–255, resized bicubically to a 50 × 50 uint8 CWD image, and loaded as a single-channel float32 tensor while retaining the 0–255 input scale. This preprocessing is applied identically to all models. The dataset construction, independent split, and input settings are summarized in Table 1.

## 3. Proposed Method

The network receives a 50 × 50 × 1 CWD image and applies a 7 × 7 convolution, BN, ELU, and 3 × 3 max pooling. Three 64-channel RepDW-NRS-ECA stages then operate at progressively reduced spatial resolution; each stage contains two RepDW units, a block residual addition, and NRS-ECA, with shrinkage strengths 0.25, 0.30, and 0.15. Global average pooling, a 64-dimensional linear layer, dropout, and a 13-class linear layer form the head. Table 2 gives the training-form architecture; the deployment form is obtained from it by exact algebraic fusion without changing nonlinear operations or block-level residual paths.

**Figure 1:**
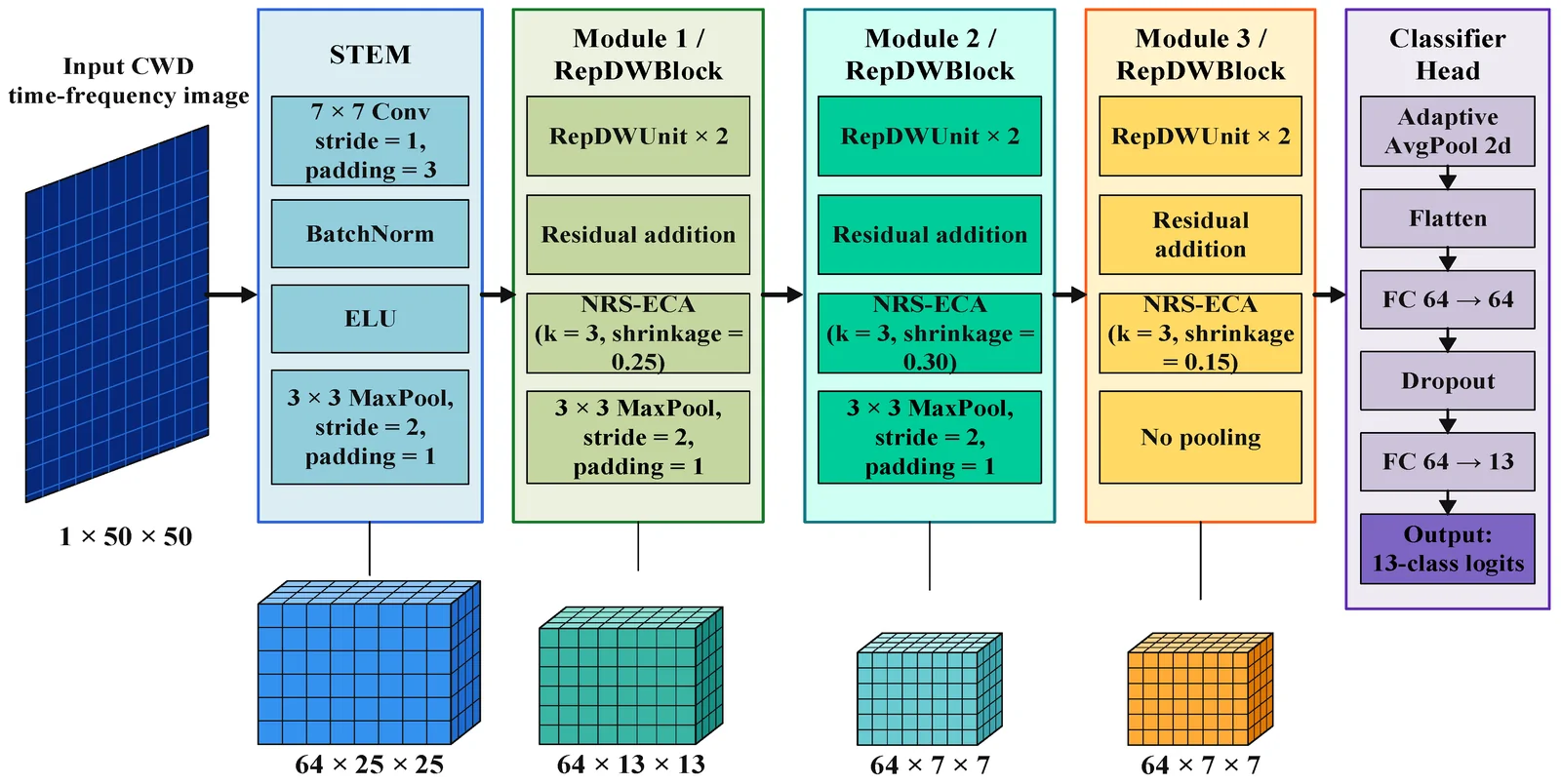
Overall workflow of the proposed method.

During the training phase, the proposed model retains the multi-branch RepDW structure to obtain more expressive time–frequency texture representations. After training, the 3 × 3, 1 × 3, 3 × 1, and identity depthwise convolution branches are first fused into RepNRS-LPI-Net-Fused-I. Then, the pointwise convolution and batch normalization layers are folded to obtain RepNRS-LPI-Net-Deploy. In the final deployment stage, RepNRS-LPI-Net-Deploy is adopted to achieve a lower parameter count and faster inference speed. The overall workflow of the proposed method is illustrated in Figure 1.

### 3.1. Multi-Branch Re-Parameterizable Depthwise Convolution Module

**Figure 2:**
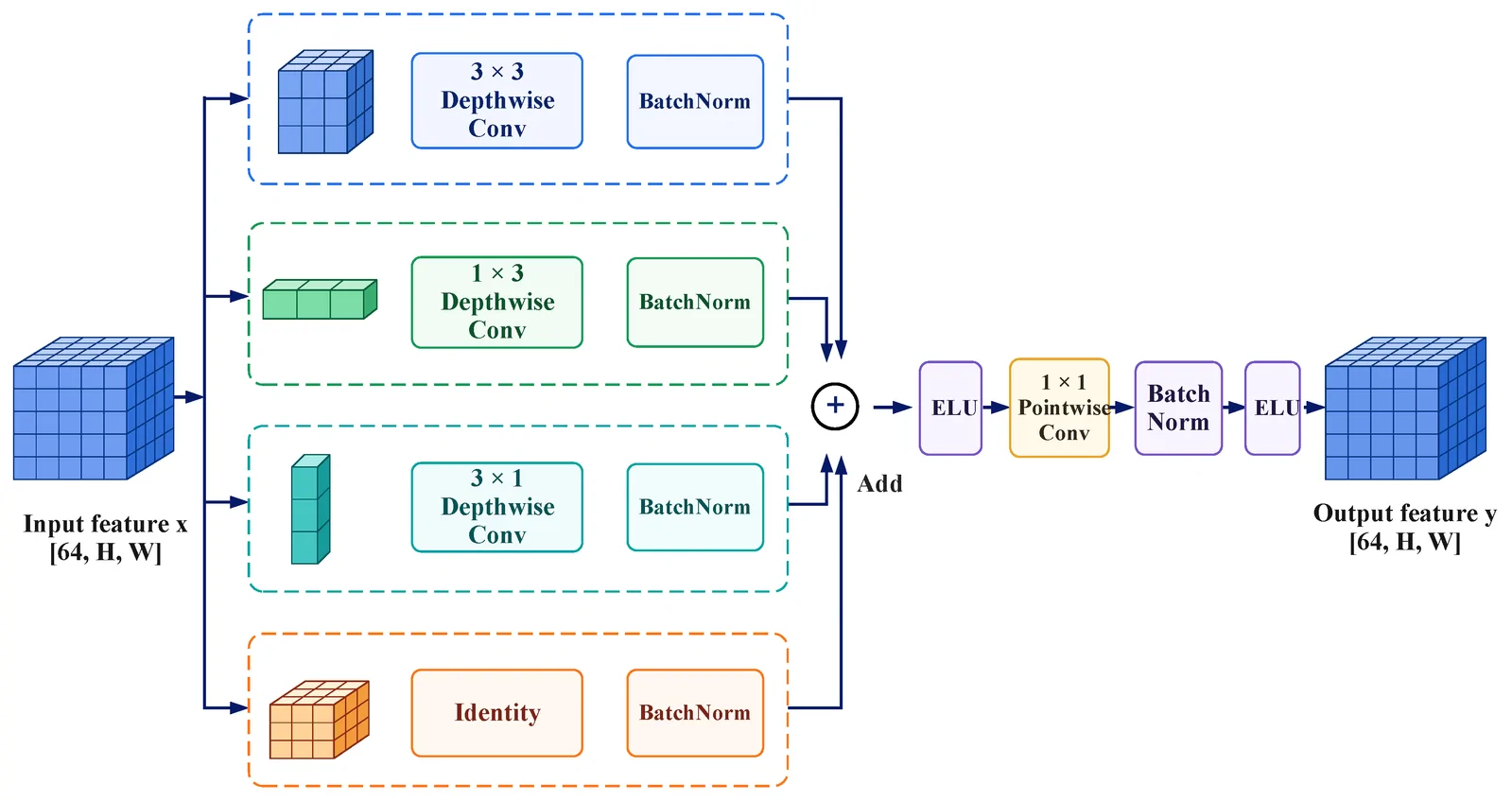
Schematic diagram of the RepDW multi-branch re-parameterizable depthwise convolution module.

RepDW is a task-oriented re-parameterizable depthwise block rather than a claim that structural re-parameterization itself is new. Its depthwise spatial filtering follows the lightweight convolution principle exemplified by MobileNets [22]. Like RepVGG, training-time branches are fused into a single deployment branch [23]; like the asymmetric convolution block in ACNet, horizontal and vertical kernels supplement a square kernel [24]. RepDW differs in using depthwise 3 × 3, 1 × 3, and 3 × 1 branches plus an identity-BN branch, followed by pointwise channel mixing, and in assigning the asymmetric branches a CWD-specific interpretation. The outputs are summed and activated by ELU before the 1 × 1 pointwise convolution, BN, and ELU. This arrangement preserves lightweight spatial filtering while exposing temporal-direction, frequency-direction, and joint time–frequency responses during training. The structure of the RepDW unit is illustrated in Figure 2.

To describe the forward feature transformation process of a RepDW unit, let X denote the input feature map. The unit can be abstracted into two consecutive steps: multi-branch depthwise convolution aggregation and pointwise channel fusion. The mathematical formulation is given as follows:

(5) $$F_{\left(DW\right)\left(X\right)}={BN}_{\left(3\times 3\right)\left({DW}_{\left(3\times 3\right)\left(X\right)}\right)}+{BN}_{\left(1\times 3\right)\left({DW}_{\left(1\times 3\right)\left(X\right)}\right)}+{BN}_{\left(3\times 1\right)\left({DW}_{\left(3\times 1\right)\left(X\right)}\right)}+{BN}_{\left(id\right)\left(X\right)}$$

(6) $$H=ELU\left(F_{\left(DW\right)\left(X\right)}\right),\;\;\;Z={BN}_{\left(PW\right)\left({PW}_{\left(1\times 1\right)\left(H\right)}\right)},\;\;\;U\left(X\right)=ELU\left(Z\right)$$

Here, DW and PW denote depthwise convolution and pointwise convolution, respectively, and $U\left(\cdot \right)\;$ represents a complete RepDW unit. Each feature extraction block contains two cascaded RepDW units and adopts a block-level residual connection. Specifically, $U_{2\left(U_{1\left(X\right)}\right)}+X$ is first computed and then fed into the NRS-ECA module. After the first two feature extraction blocks, a 3 × 3 max pooling operation with a stride of 2 is performed, whereas no pooling operation is applied after the third block.

In a CWD matrix, columns encode temporal evolution and rows encode frequency variation. The 1 × 3 depthwise branch therefore emphasizes local temporal-direction responses, the 3 × 1 branch emphasizes local frequency-direction responses, and the 3 × 3 branch captures joint time–frequency neighborhoods. The identity-BN branch preserves informative responses that should not be forced through an additional spatial filter. This interpretation is task-oriented: it does not imply that all CWD structures are axis-aligned or that asymmetric convolution is itself novel. Only these four linear in-unit branches are fused; the external block residual connection and nonlinear operations remain unchanged.

### 3.2. Noise-Aware Residual Shrinkage ECA Module

**Figure 3:**
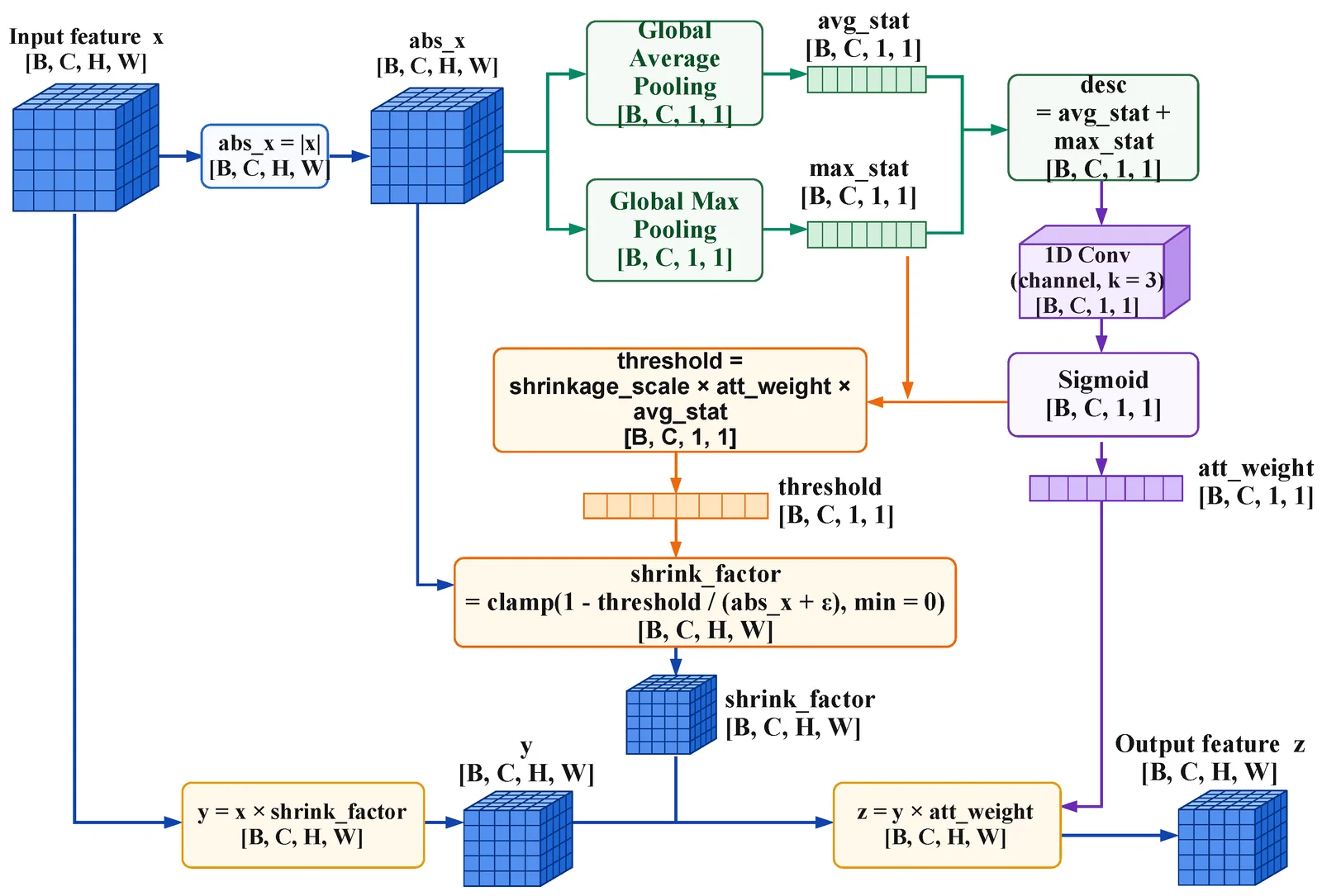
Schematic diagram of the NRS-ECA noise-aware residual shrinkage attention module.

Under low SNR, a CWD image contains both modulation-related responses and dispersed, weakly structured noise or residual cross-term responses; amplitude alone therefore cannot identify noise. NRS-ECA combines the local cross-channel interaction of ECA-Net [25] with the adaptive soft-thresholding principle of deep residual shrinkage networks [26]. Global average pooling of absolute activations summarizes persistent channel response, while global max pooling captures localized salient response. A local Conv1D models neighboring-channel dependencies and produces channel weights. These weights condition a channel-dependent threshold for soft shrinkage, attenuating weakly supported activations, after which the retained responses are recalibrated. The module is thus positioned as noise-aware shrinkage and channel recalibration, not as a guarantee that every weak response is noise. The structure of the NRS-ECA module is illustrated in Figure 3.

To describe the attention generation, threshold construction, and shrinkage-based recalibration processes of the module, let X denote the input feature map. The forward computation of NRS-ECA can be formulated as

(7) $$a=\sigma \left({Conv1D}_{3\left(GAP\left(\left|X\right|\right)+GMP\left(\left|X\right|\right)\right)}\right)$$

(8) $$T=\eta \cdot a\cdot GAP\left(\left|X\right|\right)$$

(9) $$Y=a⊙X⊙\max\left(1-\frac{T}{\left|X\right|+\delta },0\right)$$

where X denotes the input feature map. GAP and GMP represent global average pooling and global max pooling along the spatial dimensions, respectively. ${\mathrm{Conv}1D}_{3}$ denotes a one-dimensional convolution with a kernel size of 3 along the channel dimension, σ is the sigmoid function, a denotes the channel attention weights, and η is the shrinkage strength. In Equation (9), when $\left|X\right|$ at a certain position is insufficient to exceed the channel-adaptive threshold T, its shrinkage factor is truncated to zero. The remaining responses are continuously attenuated according to their amplitudes and then multiplied by the channel attention weights a. When δ approaches zero, this formulation degenerates into the conventional soft-thresholding form $a⊙\mathrm{sign}\left(X\right)⊙\max\left(\left|X\right|-T,0\right)$. The shrinkage strengths η of the three stages are set to 0.25, 0.30, and 0.15, respectively. This configuration imposes stronger constraints on noise-like responses in the low-level time–frequency textures during the first two stages, while reducing the shrinkage strength at the high-level stage to lower the risk of over-suppressing discriminative semantic features.

### 3.3. Focal Label Smoothing Loss

The dataset is balanced across all 13 classes and 31 SNR levels, so the training objective is not motivated by class-frequency imbalance. Its relevant imbalance is sample difficulty: low-SNR and structurally confusable realizations are more likely to have low true-class confidence, although Focal Loss [27] has no direct access to SNR. Cross-entropy already assigns smaller losses to easy high-confidence samples; the Focal factor further down-weights their contribution and shifts optimization toward difficult, low-confidence cases. Label Smoothing [28] softens the target distribution, reduces excessive confidence, and provides regularization. Accordingly, Focal modulation and Label Smoothing are treated here as established auxiliary training strategies and evaluated separately in the loss ablation.

(10) $$q_{i}=\left(1-\varepsilon \right)\cdot I\left(i=y\right)+\frac{\varepsilon }{C}$$

(11) $$L_{FLS}=-\alpha {\left(1-p_{y}\right)}^{\gamma }\sum_{i=1}^{C}q_{i}ln\left(p_{i}\right)$$

where C denotes the number of classes, y is the ground-truth class, $I\left(\cdot \right)$ is the indicator function, $q_{i}$ denotes the smoothed target distribution, $p_{i}$ is the Softmax probability of the (i)-th class, $p_{y}$ represents the probability of the ground-truth class, γ is the Focal modulation factor, and α is the overall scaling coefficient. In Equation (11), the Focal factor is computed from $p_{y}$ and is applied to the entire label-smoothed cross-entropy term. This differs from applying a separate Focal weight to each $p_{i}$. In the implementation, $p_{i}$ is clipped with a lower bound of $\delta =10^{-7}$ to avoid $\ln\left(0\right)$, and the samplewise losses within each mini-batch are averaged. The hyperparameters are set to γ=2.0, α=1.0, and ε=0.05. Since low-SNR and easily confused samples usually have smaller $p_{y}$, their losses are attenuated to a lesser extent. By contrast, the weights of high-confidence samples decrease as ${\left(1-p_{y}\right)}^{\gamma }$ becomes smaller.

### 3.4. Two-Stage Structural Re-Parameterization Fusion

To balance the multi-branch representation capability during training and the computational efficiency during deployment, a two-stage structural re-parameterization strategy is adopted. After training, the model is first converted from RepNRS-LPI-Net-Train to RepNRS-LPI-Net-Fused-I. In this stage, the 3 × 3, 1 × 3, and 3 × 1 depthwise Conv + BN branches, together with the identity + BN branch in each RepDW unit, are fused into a single 3 × 3 depthwise convolution with bias. Subsequently, RepNRS-LPI-Net-Fused-I is converted into RepNRS-LPI-Net-Deploy by further folding each 1 × 1 pointwise Conv + BN into a 1 × 1 convolution with bias. The two conversions are only applied to the six RepDW units in the three feature extraction blocks, while ELU, block-level residual connections, NRS-ECA, pooling layers, and the classification head remain unchanged.

(12) RepNRS-LPI-Net-Train→RepNRS-LPI-Net-Fused-I→RepNRS-LPI-Net-Deploy

(13) $$s_{b}=\frac{\gamma_{b}}{\sqrt{v_{b}+\varepsilon_{b}}},\;\;\;K_{b}^{eq}=s_{b}K_{b},\;\;\;b_{b}^{eq}=\beta_{b}+s_{b}\left(b_{b}-\mu_{b}\right)$$

(14) $$K_{DW}=K_{3\times 3}^{eq}+pad\left(K_{1\times 3}^{eq}\right)+pad\left(K_{3\times 1}^{eq}\right)+K_{id}^{eq},\;\;\;b_{DW}=b_{3\times 3}^{eq}+b_{1\times 3}^{eq}+b_{3\times 1}^{eq}+b_{id}^{eq}$$

where $K_{b}$ and $b_{b}$ denote the convolution kernel and bias of branch b, respectively. $\gamma_{b}$, $\beta_{b}$, $\mu_{b}$, $v_{b}$, and $\varepsilon_{b}$ denote the scaling coefficient, shifting coefficient, running mean, running variance, and numerical stability term of the BN layer, respectively. For the identity + BN branch, a 3 × 3 depthwise identity kernel is first constructed, where the center element of each channel is set to 1. The BN layer is then folded according to Equation (13). During fusion, the equivalent 1 × 3 and 3 × 1 kernels are zero-padded to 3 × 3 and added elementwise to the 3 × 3 and identity equivalent kernels, while the biases are directly summed. In the second stage, Equation (13) is applied again to each pointwise Conv + BN pair, yielding a 1 × 1 convolution with bias. Under inference mode with fixed BN running statistics, this transformation is algebraically equivalent, and the practical output difference only results from the floating-point computation order.

## 4. Experiments and Results Analysis

### 4.1. Experimental Setup

CWD-TFA and RepNRS-LPI-Net are compared under the same independent dataset, preprocessing, seed-2021 stratified split, and evaluation protocol. Each trained model is selected only by the highest validation accuracy, followed by one final test evaluation; the test set is not used for checkpoint selection. A separate multi-model benchmark compares LWRT [9], CWD-WR [29], CWD-TFA [30], and RepNRS-LPI-Net under a common benchmark setting. RepNRS-LPI-Net-Train is evaluated before exact fusion, while RepNRS-LPI-Net-Fused-I and RepNRS-LPI-Net-Deploy are checked for prediction equivalence and deployment efficiency.

Experiments use an Intel Core i9-13900H processor, an NVIDIA GeForce RTX 4060 Laptop GPU, 40 GB system memory, 64-bit Windows, Python 3.12.10, PyTorch 2.12.0+cu130, torchvision 0.27.0+cu130, CUDA 13.0, FP32 computation, batch size 128, and num_workers = 0. Deployment timing uses batch sizes 1, 32, 64, 128, and 256 with 30 warm-up iterations and 100 repeated measurements. Exact learned-layer complexity counts include Conv2d, Linear, and the learned NRS-ECA Conv1D; BN, activations, pooling, reductions, comparisons, and elementwise shrinkage/recalibration are excluded from the MAC total and stated separately.

### 4.2. Experimental Results and Analysis

Table 3 compares CWD-TFA and RepNRS-LPI-Net on the independent test set. RepNRS-LPI-Net achieves 79.553350% overall accuracy compared with 79.167907% for CWD-TFA, with differences of +0.385443 pp overall, +1.151515 pp at low SNR, +0.215385 pp at mid SNR, and −0.287179 pp at high SNR. The gain is largest under low SNR, where dispersed noise-like CWD responses obscure modulation-dependent ridges and textures and the directional RepDW features and NRS-ECA shrinkage are most relevant. At high SNR, both models operate near saturation, so the small difference does not support an all-SNR superiority claim.

The differences indicated in Table 3 are reported descriptively because matching sample-level CWD-TFA predictions are unavailable for a valid paired significance test. Each integer SNR from −20 to +10 dB contains 1950 test samples, and the equally sized strata yield the reported overall and range-specific macro-averages. Reporting the three SNR ranges separately shows that the measured advantage is concentrated at low SNR rather than being distributed uniformly across the test conditions.

The three execution forms of RepNRS-LPI-Net produce identical test accuracy and 100% prediction agreement. Table 3 therefore reports RepNRS-LPI-Net once, while the subsequent complexity, latency, and numerical-equivalence analyses distinguish the training and fused graphs. The unchanged decisions are expected because structural re-parameterization fuses linear convolution and batch-normalization operations algebraically without relearning model parameters.

The identical decisions isolate the role of structural re-parameterization: recognition is determined by the trained representation, while the subsequent analyses quantify how algebraic fusion changes parameter count, computation, latency, and logit-level numerical agreement.

Table 4 isolates the effects of RepDW, NRS-ECA, and the training objective. In Panel A, Full RepDW exceeds the single 3 × 3 depthwise control by 1.696 pp overall, 3.509 pp at low SNR, 1.326 pp at mid SNR, and 0.072 pp at high SNR. The larger low-SNR gain is consistent with the horizontal, vertical, and square branches capturing complementary temporal-direction, frequency-direction, and joint local CWD structures when noise obscures fine textures. In Panel B, Full NRS-ECA achieves the highest overall, low-, and mid-SNR accuracies; neither attention-only nor shrinkage-only reaches the complete module. This trend supports the combined effect of channel recalibration and adaptive attenuation of weakly supported activations, particularly under low SNR. In Panel C, Focal plus label smoothing is best overall and at low and mid SNR, whereas cross-entropy is slightly higher in the near-saturated high-SNR range. The result is consistent with Focal modulation emphasizing difficult low-confidence samples and label smoothing regularizing hard targets, while the simpler cross-entropy objective remains competitive when waveform structures are already clear.

Table 5 analyzes RepNRS-LPI-Net across quintiles of normalized Doppler (fdTp), normalized RMS multipath delay spread, and realized channel attenuation. Overall accuracy ranges from 77.014% to 83.697% across Doppler groups, 76.758% to 83.730% across multipath groups, and 79.049% to 80.017% across fading groups; the corresponding low-SNR ranges are wider for Doppler and multipath than for fading. These variations are consistent with Doppler shifts and delayed paths altering CWD ridge positions and local interference patterns, whereas the tested fading quintiles produce a narrower aggregate spread. NRS-ECA and directional CWD features can attenuate part of this modeled distortion, but the subgroup results support only the evaluated multipath, Rayleigh-fading, Doppler, and AWGN conditions and do not establish universal robustness or synchronization-error tolerance.

Table 6 compares LWRT, CWD-WR, CWD-TFA, and the three execution forms of RepNRS-LPI-Net under the benchmark setting. RepNRS-LPI-Net-Train, RepNRS-LPI-Net-Fused-I, and RepNRS-LPI-Net-Deploy achieve 81.9454% overall accuracy and 54.4362% low-SNR accuracy, compared with 81.6708% and 53.5728% for CWD-TFA, 79.2516% and 48.4227% for LWRT, and 75.1903% and 42.2591% for CWD-WR. The performance gap is most pronounced at low SNR, which is consistent with stronger CWD feature representation and noise-aware activation shrinkage being most useful when modulation structures are partially obscured.

The identical benchmark accuracies of RepNRS-LPI-Net-Train, RepNRS-LPI-Net-Fused-I, and RepNRS-LPI-Net-Deploy show that the two fusion stages do not alter recognition decisions. The benchmark trend complements the controlled CWD-TFA comparison in Table 3: both indicate that the main recognition benefit occurs under the more difficult low-SNR conditions, while the models converge as the CWD structures become clearer.

**Figure 4:**
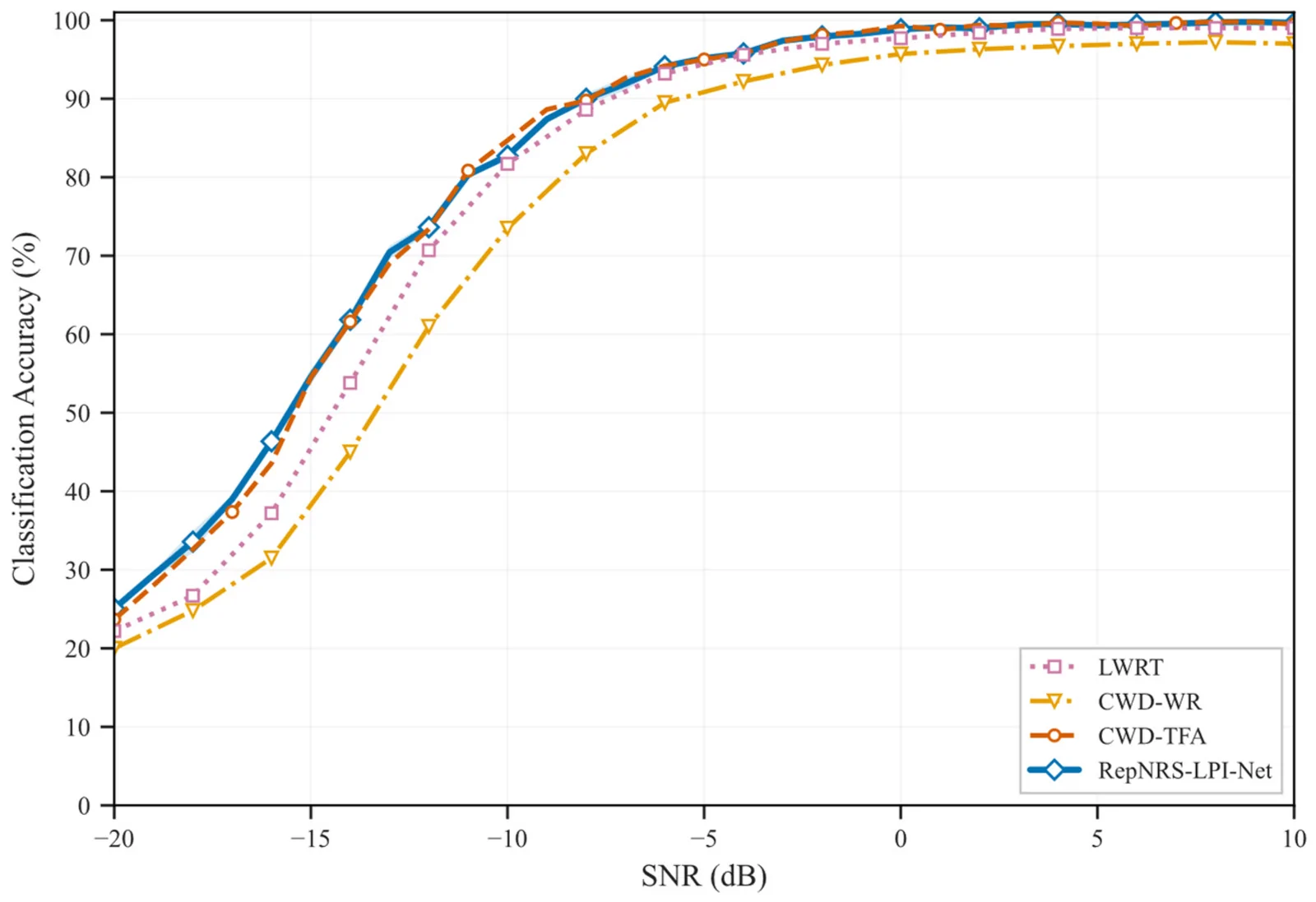
Accuracy curves of different algorithms versus SNR.

Figure 4 compares the accuracy of LWRT, CWD-WR, CWD-TFA, and RepNRS-LPI-Net from −20 to +10 dB. Accuracy rises with SNR for all four models; differences are largest at low SNR, narrow through the mid-SNR region, and approach saturation at high SNR. RepNRS-LPI-Net and CWD-TFA form the stronger curves, whereas CWD-WR remains lowest and LWRT is intermediate. This behavior is consistent with improved CWD representation and directional feature extraction preserving modulation-dependent structure under strong noise, while NRS-ECA attenuates weakly supported responses. Once the waveform trajectories and local textures become sufficiently clear at high SNR, the four models have less room to differ.

The low-SNR advantage of RepNRS-LPI-Net over CWD-TFA is more evident than the high-SNR difference, consistent with the aggregate results in Table 6. This concentration of the gain is expected because RepDW and NRS-ECA target directional texture extraction and noise-like activation suppression rather than increasing capacity indiscriminately. The independent comparison in Table 3 likewise shows improvements in overall, low-, and mid-SNR accuracy together with a small high-SNR reduction near saturation, so no all-SNR superiority claim is made.

**Figure 5:**
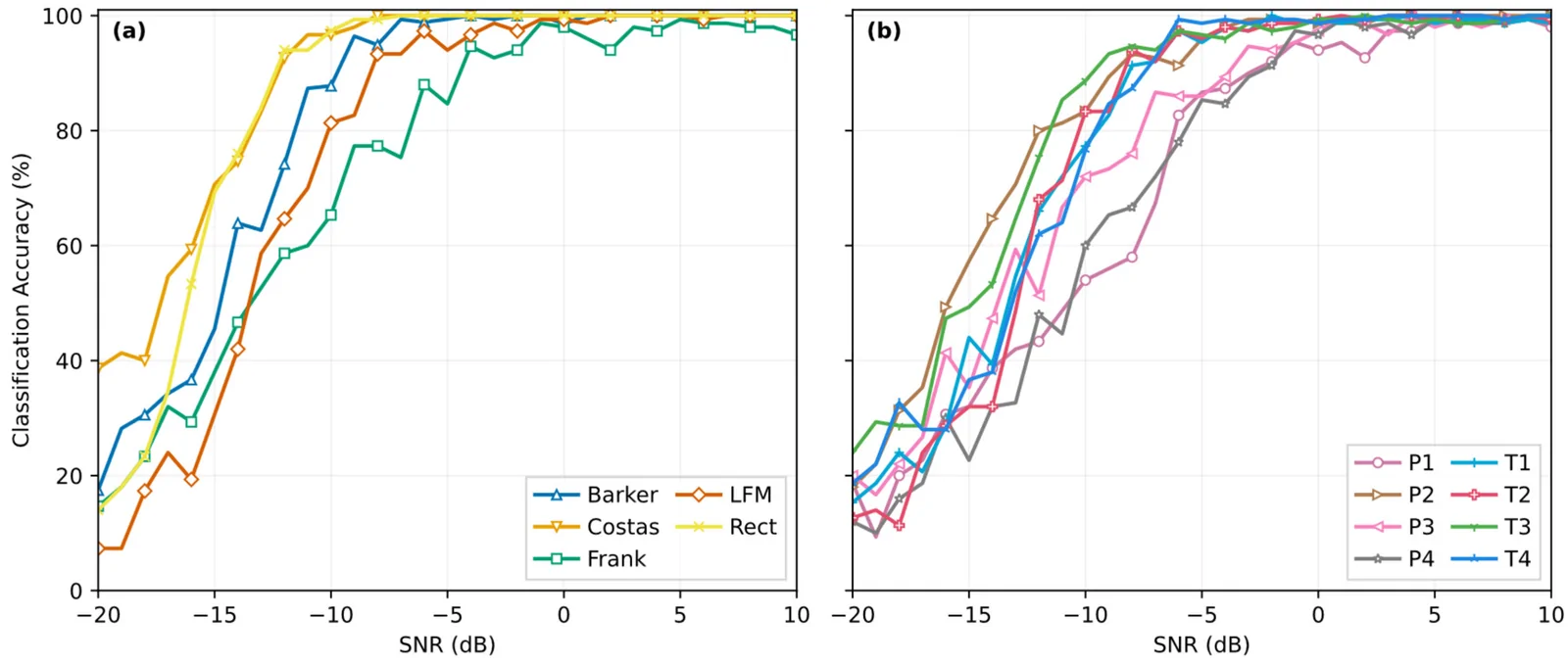
Recognition accuracy curves of 13 LPI radar waveform types under different SNRs: (**a**) common waveforms; (**b**) phase-coded waveforms.

Figure 5 presents the classwise accuracy curves of the 13 LPI waveform categories over the evaluated SNR range. Barker, LFM, Costas, and Rect rise rapidly and reach high accuracy comparatively early, whereas Frank and several polyphase- and time-coded waveforms improve more gradually and fluctuate more in the low- and mid-SNR regions. The clearer coding boundary of Barker, the linear ridge of LFM, and the distinctive frequency-hopping pattern of Costas provide strong global CWD structure. By contrast, the discrimination of Frank, P1-P4, and T1-T4 depends more heavily on fine phase-coding or local time–frequency texture, which is more readily dispersed by noise and multipath.

**Figure 6:**
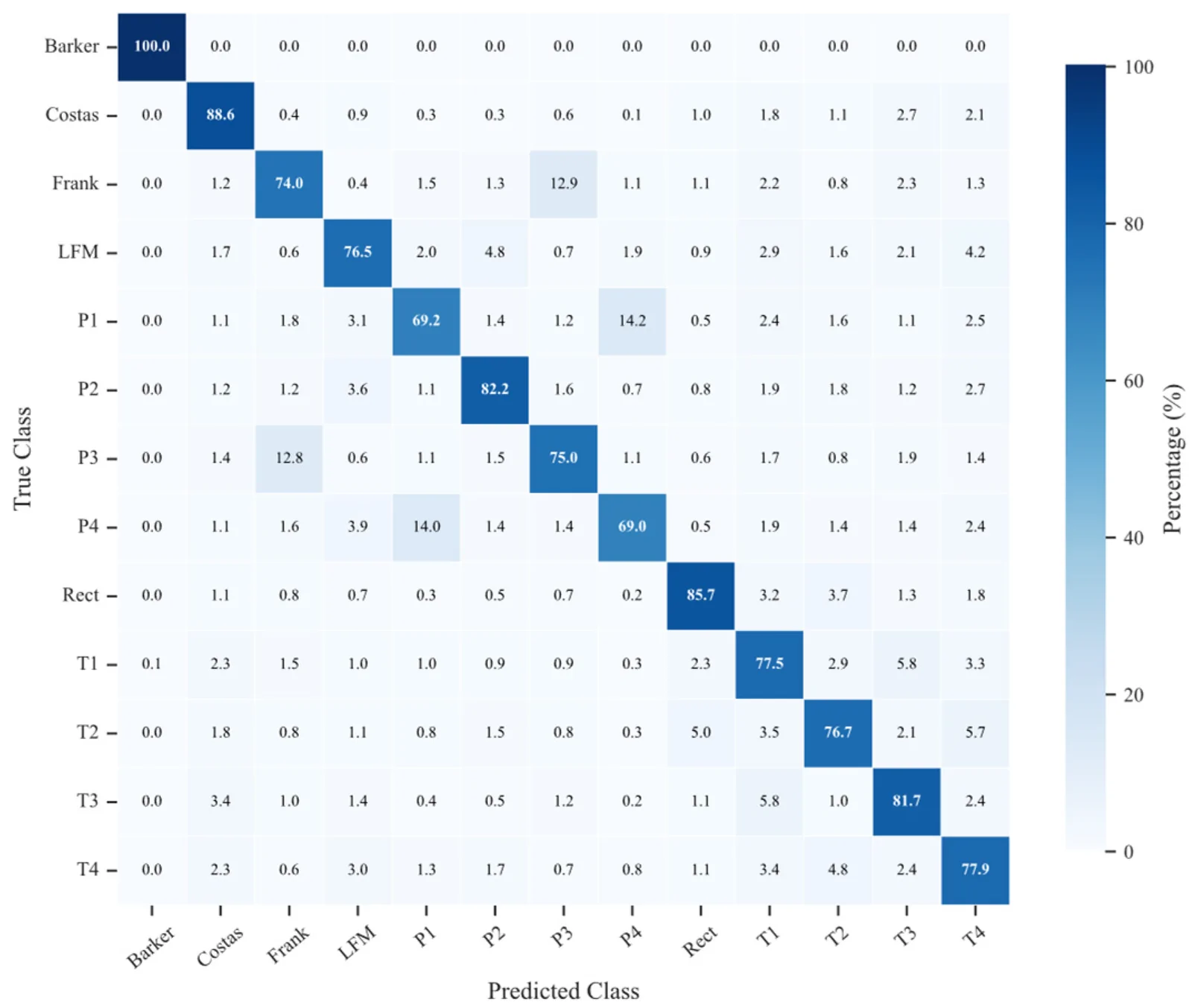
Normalized confusion matrix of the proposed method on the test set.

**Figure 7:**
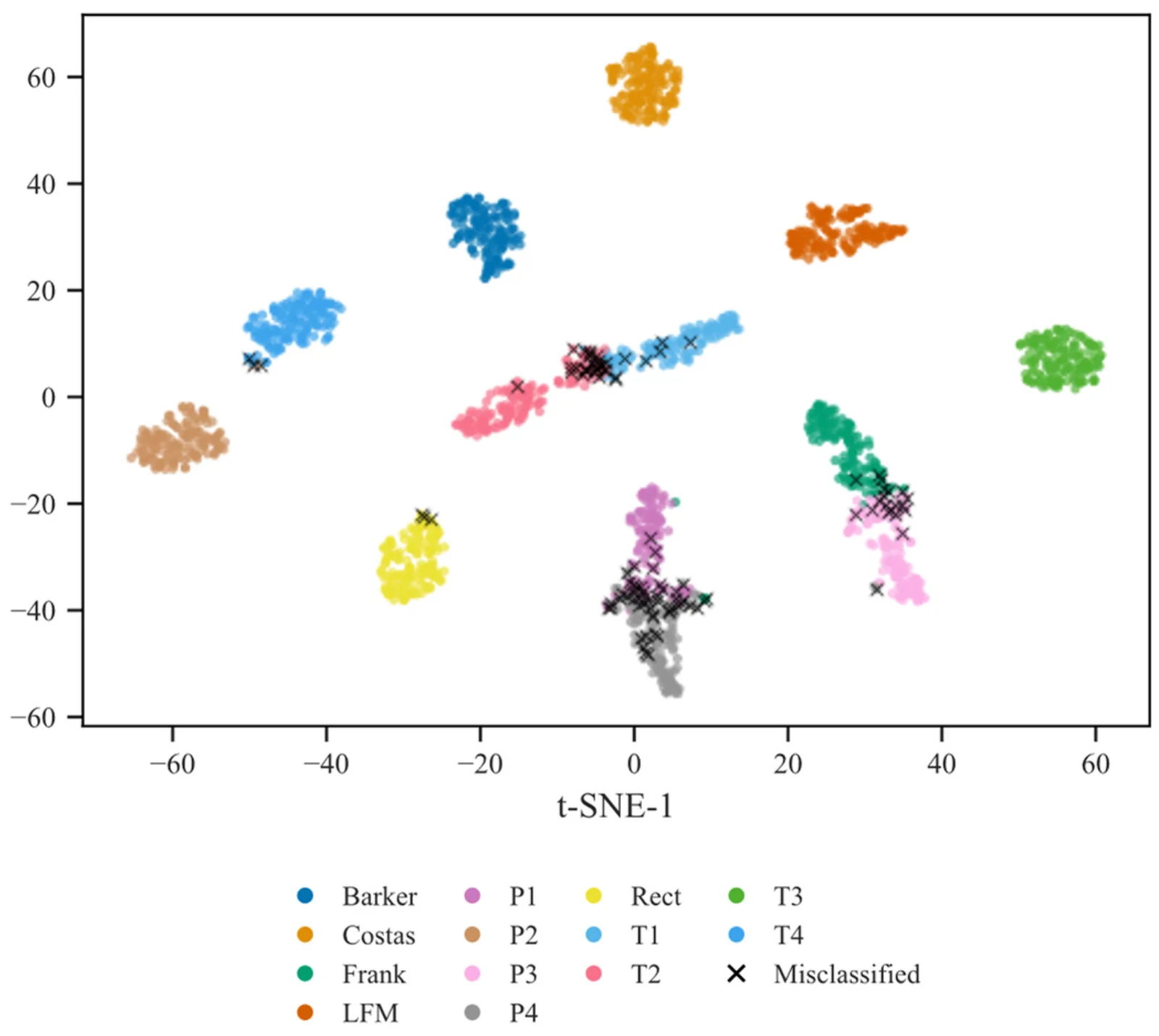
t-SNE visualization of test sample features, where colored points denote different waveform classes and black crosses denote misclassified samples.

Among the phase- and time-coded families, partial difficulty remains for P1/P4, Frank/P3, and T1/T2/T3/T4 before the curves approach saturation. Their related phase constructions can produce similar local CWD energy distributions, and low SNR further masks the subtle boundaries between coding patterns. As SNR increases, these fine structures become clearer and the class curves converge toward high accuracy; this trend is consistent with the confusion and feature-space patterns in Figure 6 and Figure 7.

Figure 6 presents the normalized confusion matrix of RepNRS-LPI-Net on the test set. The matrix is diagonally dominant, with class accuracies including 100.000% for Barker, 88.602% for Costas, 85.699% for Rect, and 82.194% for P2, while the remaining errors are concentrated among structurally related waveform families rather than distributed uniformly. This pattern indicates that the model separates waveforms with distinctive CWD ridges or coding boundaries more reliably than classes whose local phase textures overlap.

The principal confusion regions involve P1/P4, T1/T2, and Frank/P3. These pairs have related phase-coding constructions and similar local energy distributions, so low-SNR noise and modeled multipath can obscure the fine-grained CWD differences needed for separation. The nonuniform off-diagonal structure therefore reflects intrinsic waveform similarity and difficult propagation conditions rather than a uniform classification bias.

Figure 7 visualizes high-level test features using t-SNE. Most classes form compact and separated clusters, whereas partial overlaps remain between P1/P4, T1/T2, and Frank/P3, in agreement with Figure 6. The compact clusters are consistent with RepDW and NRS-ECA learning class-discriminative CWD representations, while the overlapping regions correspond to waveform families with intrinsically similar phase-coded textures.

Misclassified samples are concentrated near cluster boundaries and overlapping regions rather than spread uniformly across the projection. Low SNR and multipath weaken fine CWD textures, bringing difficult waveform representations closer together, while clearer structures form more isolated clusters. Because t-SNE is a two-dimensional qualitative projection, it illustrates feature organization but is not treated as independent quantitative proof of separability.

Table 7 compares CWD-TFA and the three execution forms of RepNRS-LPI-Net in terms of parameters, model size, MACs, and FLOPs, using one MAC = two FLOPs. The formal layer-complexity expressions are:

$$O\left(\frac{H_{out}W_{out}C_{in}C_{out}K_{h}K_{w}}{G}\right)$$

$$O\left(H_{out}W_{out}C\;K_{h}K_{w}\right)$$

$$O\left(H_{out}W_{out}C_{in}C_{out}\right)$$

Here, G denotes the number of convolution groups, and full-network complexity is the sum over stages. RepNRS-LPI-Net-Train evaluates the 3 × 3, 1 × 3, and 3 × 1 depthwise branches plus identity BN; RepNRS-LPI-Net-Fused-I evaluates one 3 × 3 depthwise branch, and RepNRS-LPI-Net-Deploy additionally folds pointwise BN.

Table 7 shows that RepNRS-LPI-Net-Train reduces parameters from 87,821 to 44,054 and MACs from 29,209,920 to 16,369,920 relative to CWD-TFA. Fusion further reduces the deployment form to 37,142 parameters and 15,722,496 MACs. These reductions follow from depthwise spatial filtering, algebraic branch fusion, and BN folding, which remove redundant learned branches and parameters at inference. BN, ELU, sigmoid, pooling, reductions, comparisons, and elementwise threshold/recalibration are excluded from the learned-layer MAC count rather than mixed with convolution and linear operations.

Table 8 compares parameter counts among LWRT, CWD-WR, CWD-TFA, and the three forms of RepNRS-LPI-Net. LWRT is largest at 1,748,573 parameters, followed by CWD-WR at 114,661 and CWD-TFA at 87,821, whereas RepNRS-LPI-Net-Train contains 44,054 parameters and the two fused forms are smaller still. The difference is primarily attributable to the large fully connected stage in LWRT and to the depthwise, compact-channel design of RepNRS-LPI-Net.

RepNRS-LPI-Net-Fused-I contains 37,910 parameters and RepNRS-LPI-Net-Deploy contains 37,142. The deployment model therefore reduces the parameter count by 57.71%, 67.61%, and 97.88% relative to CWD-TFA, CWD-WR, and LWRT, respectively. The additional reduction from the training form arises because branch fusion and BN folding convert the multi-branch training graph into a compact single-branch deployment graph without changing its decisions.

**Figure 8:**
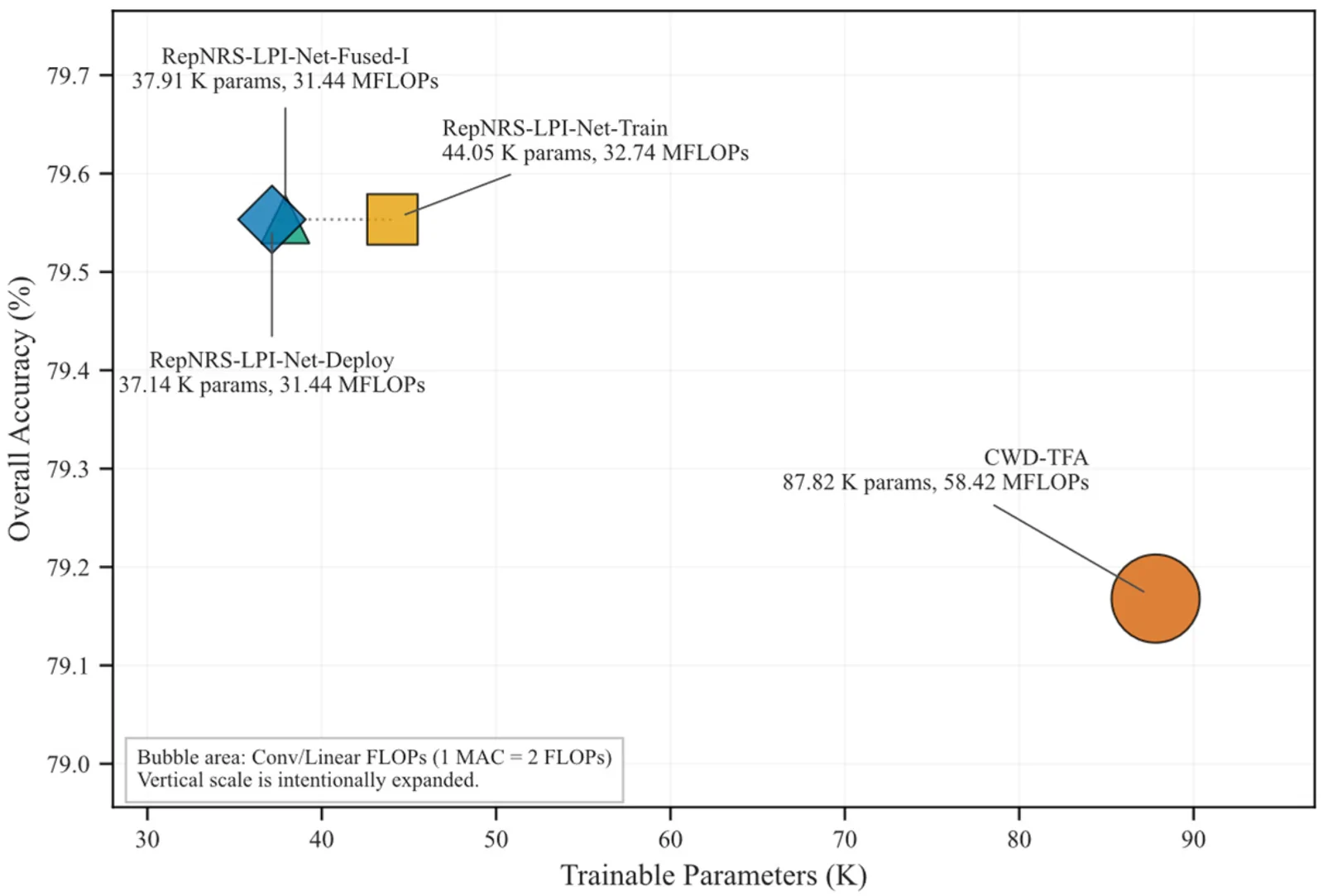
Relationship among the number of parameters, overall accuracy, and FLOPs of different models.

Figure 8 jointly compares overall accuracy, parameter count, and FLOPs for CWD-TFA, RepNRS-LPI-Net-Train, RepNRS-LPI-Net-Fused-I, and RepNRS-LPI-Net-Deploy. The RepNRS-LPI-Net variants occupy the lower-parameter and lower-FLOP region while retaining competitive accuracy, and the fused forms move further toward the lightweight side without an accuracy change. This trade-off is enabled by depthwise spatial filtering, lightweight pointwise channel mixing, and exact branch/BN fusion rather than by expanding model capacity.

In computational terms, RepNRS-LPI-Net-Train requires 32.739840 MFLOPs and both fused forms require 31.444992 MFLOPs, compared with 58.419840 MFLOPs for CWD-TFA. The reduction is consistent with replacing standard spatial convolution by depthwise filtering and removing training-time branch overhead through structural re-parameterization; elementwise operations excluded by the Table 7 convention are not included in these values.

Table 9 compares median RTX 4060 FP32 forward latency for CWD-TFA and the three forms of RepNRS-LPI-Net at batch sizes 1, 32, 64, 128, and 256. RepNRS-LPI-Net-Train is slower because its multi-branch graph retains additional operator and normalization overhead, whereas RepNRS-LPI-Net-Deploy is the fastest measured graph at every listed batch size, including 1.482850 ms/image at batch 1 and 0.047499 ms/image at batch 128. Per-image latency generally decreases as batching improves GPU parallel utilization, while the exact value remains hardware- and implementation-dependent.

The speed gain after fusion is consistent with reducing branch scheduling, normalization, and intermediate-memory traffic. The latency measurements complement the exact MAC/FLOP analysis in Table 7; they are reported with device, precision, warm-up, repeat, and batch-size conditions and are not generalized to other platforms.

Table 10 verifies numerical consistency for RepNRS-LPI-Net-Train to RepNRS-LPI-Net-Fused-I and then to RepNRS-LPI-Net-Deploy over the complete test set. Both transitions preserve every predicted class and produce zero accuracy difference; the maximum absolute logit errors are 1.859665 × 10^−5^ and 1.001358 × 10^−5^, respectively. The small nonzero logit differences are consistent with floating-point operation order.

The 100% prediction agreement is expected because branch fusion and BN folding are algebraic transformations of fixed linear operations and do not relearn parameters. Table 10 therefore supports deployment of the compact graph without attributing any accuracy change to fusion, while the reported errors avoid implying bitwise-identical logits.

Table 11 summarizes the recognition-complexity trade-off of RepNRS-LPI-Net-Deploy relative to CWD-TFA. Overall, low-SNR, and mid-SNR accuracies increase by 0.385443, 1.151515, and 0.215385 pp, respectively, while high-SNR accuracy decreases by 0.287179 pp. At the same time, exact prediction agreement is maintained and parameters, model size, and MACs decrease by 57.71%, 58.72%, and 46.17%. The concentration of the accuracy gain at low SNR is consistent with directional CWD feature extraction and noise-aware shrinkage, whereas the deployment reductions arise from depthwise convolution and exact fusion.

Taken together, Table 3, Table 4, Table 5, Table 6, Table 7, Table 8, Table 9, Table 10 and Table 11 and Figure 4, Figure 5, Figure 6, Figure 7 and Figure 8 show that RepNRS-LPI-Net improves overall, low-SNR, and mid-SNR recognition, exhibits a small high-SNR reduction near saturation, and reduces deployment complexity under the modeled channel conditions. The controlled ablations support the contributions of complete RepDW, NRS-ECA, and Focal plus label smoothing, while the latency and fusion analyses show that the trained representation can be converted into an efficient deployment graph without changing test decisions.

## 5. Conclusions

This paper presents RepNRS-LPI-Net as a task-oriented co-design of directional CWD feature extraction, noise-aware shrinkage/channel recalibration, and exact lightweight deployment. On the independent dataset, the method improves overall, low-SNR, and mid-SNR accuracy while showing a small high-SNR decrease near saturation; its fused deployment form substantially reduces parameters, model size, MACs, and measured RTX 4060 latency without changing test decisions. The systematic ablation further confirms that the complete RepDW, NRS-ECA, and combined Focal and label-smoothing configurations provide the strongest overall and low-SNR performance among their respective controlled variants. The conclusions are limited to synthetic waveforms under the modeled multipath, Rayleigh-fading, Doppler, and AWGN conditions. Explicit synchronization mismatch, over-the-air hardware effects, and real RF measurements were not evaluated and remain important future work.

## Figures and Tables

**Table 1:** Dataset construction, independent split, and input settings.

Item	Value
Classes/SNR grid	13/−20 to +10 dB, 1 dB interval (31 levels)
Independent realizations	1000 per class × SNR; 403,000 total
Per-stratum split	700 train/150 validation/150 test
Total split	282,100/60,450/60,450
Split unit	Independent waveform + channel + Doppler + AWGN realization
Test SNR bins	Low 21,450; Mid 19,500; High 19,500
Modeled channel	6-path Rayleigh; Doppler; complex AWGN
CWD/network input	50 × 50 uint8; float32 on 0–255 scale

**Table 2:** Core architecture of RepNRS-LPI-Net (training form).

Stage/Block	Output Size	Core Operation	Kernel/Configuration	Stride/Groups/Channels
Input	1 × 50 × 50	CWD image	50 × 50 single-channel input	1 channel
Stem	64 × 25 × 25	Conv2d + BN + ELU; MaxPool2d	7 × 7 convolution; 3 × 3 pooling	stride 1 then 2; groups 1; 1 -> 64
Stage 1	64 × 13 × 13	RepDW block (2 sequential units) + residual + NRS-ECA + pooling	RepDW: 3 × 3, 1 × 3, 3 × 1, identity; Conv1D k = 3; shrinkage s = 0.25; 3 × 3 pool	stride 1; DW groups 64; 64 channels; pool stride 2
Stage 2	64 × 7 × 7	RepDW block (2 sequential units) + residual + NRS-ECA + pooling	RepDW: 3 × 3, 1 × 3, 3 × 1, identity; Conv1D k = 3; shrinkage s = 0.30; 3 × 3 pool	stride 1; DW groups 64; 64 channels; pool stride 2
Stage 3	64 × 7 × 7	RepDW block (2 sequential units) + residual + NRS-ECA	RepDW: 3 × 3, 1 × 3, 3 × 1, identity; Conv1D k = 3; shrinkage s = 0.15	stride 1; DW groups 64; 64 channels
Classifier	64	Global average pooling; Linear; Dropout	global 7 × 7 pooling; 64-unit FC; *p* = 0.5	64 channels
Output	13	Linear logits	64 -> 13	13 classes

**Table 3:** Same-dataset comparison of CWD-TFA and RepNRS-LPI-Net.

Model	Overall Acc	Low SNR Acc	Mid SNR Acc	High SNR Acc
CWD-TFA	79.167907%	47.986014%	93.102564%	99.533333%
RepNRS-LPI-Net	79.553350%	49.137529%	93.317949%	99.246154%
Delta (RepNRS-CWD-TFA)	+0.385443 pp	+1.151515 pp	+0.215385 pp	−0.287179 pp

**Table 4:** Controlled systematic ablation on the independent dataset (%).

Panel	Variant	Overall	Low	Mid	High
A. RepDW	Single 3 × 3 DW	77.857	45.629	91.992	99.174
A. RepDW	Full RepDW	79.553	49.138	93.318	99.246
B. NRS-ECA	No NRS-ECA	77.826	45.476	91.941	99.297
B. NRS-ECA	Attention-only	78.025	46.098	92.067	99.103
B. NRS-ECA	Shrinkage-only	78.152	46.410	92.144	99.077
B. NRS-ECA	Full NRS-ECA	79.553	49.138	93.318	99.246
C. Loss	Cross-entropy	78.789	47.142	92.949	99.441
C. Loss	Focal	77.763	46.224	91.215	99.005
C. Loss	Label smoothing	78.440	46.844	92.272	99.364
C. Loss	Focal + smoothing	79.553	49.138	93.318	99.246

**Table 5:** Test-set subgroup robustness across physical-severity quintiles.

Impairment	Scope	Quintile Count	RepNRS Acc. Range
Doppler	Overall	12,090	77.014–83.697%
Doppler	Low SNR	4200–4345	43.797–59.238%
Multipath	Overall	12,090	76.758–83.730%
Multipath	Low SNR	4230–4373	42.104–62.340%
Fading	Overall	12,090	79.049–80.017%
Fading	Low SNR	4227–4374	48.379–50.160%

**Table 6:** Benchmark comparison of overall and SNR-range-specific accuracy among different models.

Model	Overall Acc	Low SNR Acc	Mid SNR Acc	High SNR Acc
LWRT	79.2516%	48.4227%	93.6200%	98.7950%
CWD-WR	75.1903%	42.2591%	89.8300%	96.7750%
CWD-TFA	81.6708%	53.5728%	94.9280%	99.4648%
RepNRS-LPI-Net-Train	81.9454%	54.4362%	94.7911%	99.4861%
RepNRS-LPI-Net-Fused-I	81.9454%	54.4362%	94.7911%	99.4861%
RepNRS-LPI-Net-Deploy	81.9454%	54.4362%	94.7911%	99.4861%

**Table 7:** Exact parameter, model-size, and learned-layer complexity comparison.

Model	Parameters	Model Size (MiB)	MACs	FLOPs
CWD-TFA	87,821	0.344433	29,209,920	58,419,840
RepNRS-LPI-Net-Train	44,054	0.183426	16,369,920	32,739,840
RepNRS-LPI-Net-Fused-I	37,910	0.148087	15,722,496	31,444,992
RepNRS-LPI-Net-Deploy	37,142	0.142181	15,722,496	31,444,992

**Table 8:** Comparison of the number of parameters among different models.

Model	Number of Parameters	Parameter Change Relative to CWD-TFA	Parameter Change Relative to CWD-WR	Parameter Change Relative to LWRT
LWRT	1,748,573	-	-	-
CWD-WR	114,661	-	-	-
CWD-TFA	87,821	-	-	-
RepNRS-LPI-Net-Train	44,054	−49.84%	−61.58%	−97.48%
RepNRS-LPI-Net-Fused-I	37,910	−56.83%	−66.94%	−97.83%
RepNRS-LPI-Net-Deploy	37,142	−57.71%	−67.61%	−97.88%

**Table 9:** Median RTX 4060 FP32 forward latency (ms/image; 30 warm-ups, 100 repeats).

Batch Size	CWD-TFA	RepNRS-LPI-Net-Train	RepNRS-LPI-Net-Fused-I	RepNRS-LPI-Net-Deploy
1	1.695000	2.870300	1.717500	1.482850
32	0.058961	0.094723	0.050108	0.045461
64	0.058748	0.082584	0.045894	0.044819
128	0.067010	0.086583	0.049701	0.047499
256	0.074785	0.096754	0.058116	0.055473

**Table 10:** Complete-test-set numerical equivalence of two-stage structural re-parameterization.

Fusion Stage	Max abs. Error	Mean abs. Error	Agreement	Accuracy diff.
Train -> RepNRS-LPI-Net-Fused-I	1.859665 × 10^−5^	8.003296 × 10^−7^	100.0000%	0.0000 pp
RepNRS-LPI-Net-Fused-I -> RepNRS-LPI-Net-Deploy	1.001358 × 10^−5^	8.185953 × 10^−7^	100.0000%	0.0000 pp

**Table 11:** Summary of RepNRS-LPI-Net-Deploy relative to CWD-TFA.

Metric	Deploy vs. CWD-TFA
Overall accuracy	+0.385443 pp
Low-SNR accuracy	+1.151515 pp
Mid-SNR accuracy	+0.215385 pp
High-SNR accuracy	−0.287179 pp
Parameters	−57.71%
Model size	−58.72%
MACs	−46.17%

## Data Availability

The original contributions presented in this study are included in the article. Further inquiries can be directed to the corresponding author.
